# Early-warning prediction of visceral leishmaniasis mortality using a multivariate STL–deep learning hybrid approach on 20 years of monthly time series

**DOI:** 10.3389/fpubh.2026.1754966

**Published:** 2026-03-26

**Authors:** Fathelrhman El Guma, Maaweya Awadalla, Halah Z. Al Rawi, Bashayer Saeed, Huda M. Alshanbari, Alshaikh A. Shokeralla, Bandar Alosaimi

**Affiliations:** 1 Department of Mathematics, Faculty of Science, Al-Baha University, Al-Aqiq, Saudi Arabia; 2 Research Center, King Fahad Medical City, Riyadh Second Health Cluster, Riyadh, Saudi Arabia; 3 Department of Mathematical Sciences, College of Science, Princess Nourah Bint Abdulrahman University, Riyadh, Saudi Arabia

**Keywords:** climate-sensitive diseases, deep learning, epidemiological forecasting, multivariate time series, STL decomposition, visceral leishmaniasis

## Abstract

**Introduction:**

Visceral leishmaniasis (VL) is a preventable disease, but continues to cause mortality in Sudan, with transmission dynamics and potentially fatal outcomes strongly affected by local environmental conditions.

**Methods:**

This research presents an innovative hybrid forecasting framework that amalgamates Seasonal-Trend decomposition using Loess (STL) with four sophisticated models: Gaussian Process Regression (GPR), Long Short-Term Memory (LSTM), Temporal Pattern Attention-LSTM (TPA-LSTM), and Light Gradient Boosting Machine (LightGBM), to forecast climate-induced multivariate VL mortality in Gedaref State, Sudan. Twenty years of monthly time series data from 2002 to 2022 were used, integrating VL mortality counts with meteorological variables such as precipitation, temperature, and relative humidity. The model’s performance was evaluated using MAE, RMSE, MAPE, *R*^2^, Willmott Index, and PBIAS.

**Results:**

Among the models, STL-LightGBM exhibited the best predictive accuracy (*R*^2^ = 0.9491), whereas the deep learning approaches inadequately captured non-linearities, long-term dependencies, and seasonal changes. In this work, we concentrate on mortality prediction, hence directly contributing to a large research gap that has not been tackled by other works, which have been focused on the prediction of VL incidence.

**Discussion:**

This proposed system has great potential in being an early-warning tool, which could be used to predict death surges and the seasonal variation, contribute by distributing pharmaceuticals and diagnostic devices, and help prepare rural health systems. These findings demonstrate the great potential of hybrid decomposition-learning models in the prediction of NTDs in regionally specific, resource-limited and climate-dependent regions.

## 1 Introduction

Visceral leishmaniasis (VL), often known as kala-azar, is a severe parasitic disease caused by Leishmania parasites, primarily the Leishmania donovani complex, and transmitted through the bites of infected female sandflies. The disease is widespread in tropical and subtropical regions, particularly in East Africa and South Asia, where it remains a significant public health concern (1, 2, 42, 43). If left untreated, VL is almost always fatal, making early detection and timely intervention critical for preventing mortality. Leishmaniasis occurs in three main clinical forms: cutaneous, mucocutaneous, and visceral, of which VL is the most severe and life-threatening (1, 2).

Notwithstanding global control efforts, visceral leishmaniasis remains a major public health problem, with an estimated 50,000–90,000 new cases reported annually worldwide (3). However, these figures likely underestimate the true burden due to underdiagnosis and underreporting in many endemic regions (4). If left untreated, VL is almost always fatal, with case fatality rates exceeding 95% among symptomatic individuals according to the World Health Organization (WHO) (41). This persistent fatality burden highlights the importance of anticipating periods of elevated mortality risk rather than focusing solely on disease incidence.

In VL-endemic East Africa, the risk of death from untreated disease is amplified by limited access to health services, malnutrition, poverty, and co-morbid conditions such as HIV infection, malnutrition, and other chronic diseases, which accelerate clinical deterioration (44). Sudan, particularly Gedaref State in Eastern Sudan, represents a major endemic focus of VL burden (5–8), where delayed detection and limited treatment access continue to result in preventable deaths in rural and resource-constrained communities.

Climatic variability significantly influences VL transmission patterns by affecting sandfly survival, nesting sites, biting behavior, and seasonal abundance. These ecological consequences can increase transmission intensity, leading to a higher incidence of severe infections and, subsequently, heightened mortality among vulnerable groups.

The persistently high case fatality associated with untreated VL underscores the importance of early diagnosis and timely intervention in these settings. It also highlights the need for predictive early-warning systems capable of identifying periods of elevated mortality risk and guiding targeted public health responses (9, 10).

Numerous studies have investigated the utilization of machine learning and time-series methodologies for forecasting the dynamics of visceral leishmaniasis in endemic areas. These studies have illustrated the capacity of meteorological and epidemiological data to enhance illness forecasting and public health planning. Climate-informed models have been employed to predict leishmaniasis outbreaks in Brazil and Sri Lanka, underscoring the impact of humidity, rainfall, and temperature on disease transmission dynamics (15, 16, 45, 46). Comparable methodologies in South Asia, Morocco, and Sudan have demonstrated that hybrid machine learning and statistical models can enhance short-term predictions of VL prevalence (11–14).

Nevertheless, the majority of current research has focused on forecasting incidence rather than mortality, with many studies relying on univariate or restricted multivariate models. Moreover, predictive performance has frequently been assessed by random data partitioning instead of validation suitable for time-series, potentially leading to an overestimation of forecasting capability in temporally organized epidemiological data. Moreover, although climatic variables have been included in numerous studies, their impact has predominantly been analyzed in terms of transmission or case counts rather than mortality outcomes.

Table 1 summarizes key studies on the forecasting of visceral leishmaniasis, highlighting their methodological frameworks, geographic scope, and principal conclusions. Nevertheless, the majority of prior research has focused on forecasting incidence rather than mortality and has frequently used univariate or restricted multivariate variables, thereby inflating predictive accuracy in temporally sequenced epidemiological data. Moreover, climate variability has predominantly been analyzed in terms of transmission and case numbers rather than mortality. Thus, there is an urgent need for climate-driven multivariate time-series methodologies to predict mortality and deliver actionable early-warning indicators in high-burden endemic regions such as eastern Sudan.

**Table 1:** Comparative summary of previous studies on VL forecasting.

Study	Location	Focus	Methods	Outcome	Reference
Time series analysis of leishmaniasis incidence in Sri Lanka	Sri Lanka	Incidence forecasting	Time Series (SARIMA), Humidity analysis	Seasonal incident patterns	Wijerathna, T., and Gunathilaka, N. (34)
Modeling climate change impacts on VL	India (Bihar)	Incidence and climate	ML models, Climate data	Climate-driven incidence prediction	Kumar et al. (11)
Incidence and prediction of leishmaniasis cases	Morocco	CL forecasting	ARIMA models	Climate-driven incidence	Hakem et al. (12)
Prediction of visceral leishmaniasis	Sudan	Incidence forecasting	LSTM, SARIMA	LSTM outperformed SARIMA	El Guma (13)
Prediction of Visceral Leishmaniasis Incidences Utilizing Machine Learning Techniques	Sudan	Incidence forecasting	Machine Learning Techniques	Showed ML feasibility for VL incidence prediction in Sudan	Guma et al. (14)
Predicting the number of visceral leishmaniasis	China	Incidence forecasting	ARIMA-EGARCH hybrid	Volatility-adjusted predictions	Li et el (35)
Predicting leishmaniasis outbreaks in Brazil using ML	Brazil	Incidence forecasting	ML (Random Forest, SVM), Meteorological data	Short-term incidence prediction	Donizette et al. (36)
The Granger Causality Analysis of Climatic Factors on VL	Iran	Climate-VL association	Granger Causality, Time Series	Climatic Drivers’ identification	Hamta, et al. (37)
Integrating AI for infectious disease prediction	Pakistan	Incidence forecasting	Hybrid ANN-XGBoost	Improved accuracy over ARIMA	Niu et al. (38)
Modeling of Leishmaniasis Infection	Morocco	CL forecasting	VAR, VECM, GLM, Markov Switching	Model comparison	Badaoui et al. (39)
Analysis of leishmaniasis military	Iran	CL forecasting	ARIMA	Incidence modeling	Tadayonfar et al. (40)

This research provides multiple contributions to the epidemiological prediction of visceral leishmaniasis. First, it establishes a climate-dependent, multivariate mortality forecasting framework that combines STL analysis with machine learning and deep learning models, facilitating the accurate modeling of seasonal, trending, and residual components of visceral leishmaniasis dynamics.

The study focuses specifically on mortality rather than morbidity, offering a practical perspective for public health planning in endemic, resource-limited areas.

Third, it integrates delayed climatic and epidemiological signals (*t* − 1, *t* − 2, *t* − 3) to represent biologically probable transmission pathways and delayed vector responses to environmental changes.

Finally, this work is the first long-term (20-year) study of climate-related mortality forecasting for visceral leishmaniasis in East Africa, providing a practical framework for early prediction applicable in resource-limited settings.

## 2 Methods

This study proposes a hybrid Seasonal-Trend decomposition procedure based on Loess (STL), integrated with advanced deep and machine learning models, to forecast visceral leishmaniasis (VL) mortality using climate-sensitive multivariate time-series data from Gedaref, Sudan.

### 2.1 Study area and data collection

**Figure 1:**
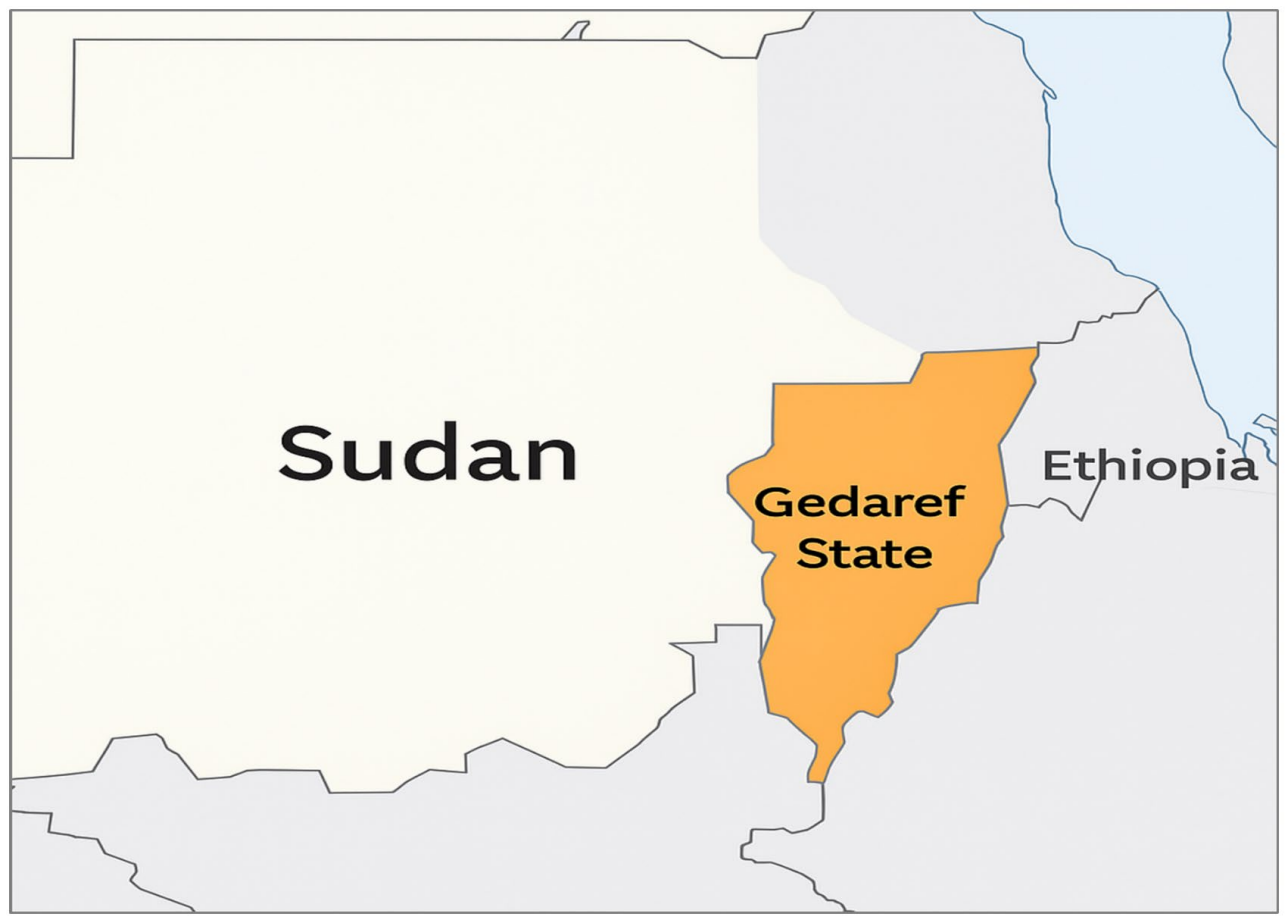
Geographic position of Gedaref State in eastern Sudan together with its administrative borders.

Gedaref State is situated in eastern Sudan and shares a border with Ethiopia, as depicted in Figure 1. It is a principal endemic focus of visceral leishmaniasis, characterized by a semi-tropical climate and a distinct rainy season from June to September, with annual precipitation ranging from 700 to 900 mm. The environmental circumstances significantly affect sandfly ecology and the dynamics of disease transmission.

Monthly statistics on VL mortality and incidence (2002–2022) were acquired from the Sudanese Ministry of Health. Climatic factors such as precipitation, temperature, and relative humidity were sourced from the Sudan Meteorological Authority for the identical timeframe. The complete dataset consisted of 240 monthly observations. Gedaref has an estimated population of around 2.2 million; hence, mortality was assessed using absolute counts, with incidence incorporated as a covariate to represent exposure dynamics.

Epidemiological data: Monthly counts of VL mortality (deaths) for the period January 2002–December 2022 (inclusive), obtained from the Sudanese Ministry of Health. Monthly VL incident (reported cases) for the same period were also used as an explanatory variable.

- Climatic variables: Monthly cumulative rainfall (mm), monthly mean temperature (°C), and monthly mean relative humidity (%) obtained from the Sudan Meteorological Authority for the same temporal window.

All-time series are aligned to monthly time steps. The final multivariate dataset consists of T = 240 monthly observations (January 2002–December 2022) across multiple epidemiological and climatic variables (mortality, incidence, rainfall, temperature, humidity plus engineered predictors described below).

During the study period, Gedaref State has an estimated population of over 2.2 million residents. The population size remained largely constant throughout the study period; therefore, mortality was assessed using absolute counts rather than population-adjusted rates. Incidence was used as a covariate to partially account for population-level exposure dynamics.

### 2.2 Data preprocessing

The raw data underwent a structured preprocessing pipeline to prepare robust inputs for decomposition and modeling.

#### 2.2.1 Missing values and quality control

Time stamps were verified and aligned. Missing monthly observations were infrequent; imputation was performed using linear interpolation followed by Kalman smoothing to respect temporal continuity and avoid introducing abrupt artifacts (15). Isolated extreme outliers were inspected and minorized only if inconsistent with source records.

#### 2.2.2 Scaling and normalization

To facilitate model training and maintain comparable scales, continuous variables were scaled to the [0,1] interval using Min–Max normalization. For models that benefit from standardization (e.g., GPR), standardized variants (zero mean, unit variance) were prepared as needed. To ensure reproducibility, all preparation steps were performed in a controlled Python environment, using the same random settings and consistent transformation methods for both the training and test data.

Table 2 depicted the descriptive statistics reveal significant diversity in both epidemiological and meteorological variables. The incidence and death of VL have right-skewed distributions, indicating transmission dynamics driven by outbreaks rather than stable endemic levels. Rainfall demonstrates significant unpredictability and pronounced skewness, aligning with the episodic precipitation patterns observed in eastern Sudan. Temperature and relative humidity exhibit modest variability with consistent averages, reinforcing their significance as climatic factors in the multivariate forecasting model.

**Table 2:** Descriptive statistics of epidemiological and climatic variables (Gedaref, Sudan; 2002–2022).

Variable	Description	Mean	Std	Min	25%	Median	75%	Max	Skewness	Kurtosis
VL deaths	Monthly mortality counts	8.45	6.56	0	3.75	7.00	11.25	30	1.06	0.46
VL cases	Monthly reported incidence	268.82	167.12	67	145.00	217.50	332.00	876	1.27	1.30
Temperature (°C)	Monthly mean air temperature	27.94	2.26	22.13	26.04	27.75	29.65	33.03	0.11	−0.78
Relative humidity (%)	Monthly mean relative humidity	57.08	12.00	38.26	48.08	53.13	66.15	83.12	0.61	−0.82
Rainfall (mm)	Monthly cumulative rainfall	50.78	78.36	0	0.04	9.63	73.19	470.56	2.00	4.30

#### 2.2.3 Stationarity and seasonality checks

Each series was visually inspected (seasonal plots, ACF/PACF) and formally tested with the Augmented Dickey–Fuller (ADF) test (16). Where strong non-stationarity remained in residuals after STL, limited differencing (first order) was examined only to support specific models; however, the principal pipeline relies on decomposition to isolate non-stationary trend and seasonal components rather than aggressive differencing.

#### 2.2.4 Feature engineering

We constructed a suite of derived predictors designed to capture delayed climatic effects and inter-variable interactions commonly observed in vector-borne disease dynamics:

- Lag features: For each climatic and incidence variable, lagged values at *t* − 1, *t* − 2, and *t* − 3 months were created to represent the delayed effects on VL mortality. Lag periods of 1 to 3 months were chosen based on epidemiological studies demonstrating a delayed response of sandflies to climate variability and the incubation-related development from infection to severe illness outcomes (17). Prior research in East Africa has shown that precipitation and humidity impact sandfly reproductive conditions and transmission intensity, whereas temperature influences vector viability and parasite development (18, 19). Integrating lag structures facilitates biologically credible modeling of transmission channels and mortality dynamics, enhancing the capacity of time-series models to comprehend delayed climate-disease interactions (20).

The intended lagged predictors were integrated into the STL decomposition and subsequent machine-learning models to improve temporal feature learning and predictive precision.
Rolling statistics: 3-month and 6-month moving averages and moving standard deviations were computed for rainfall and humidity to capture short- to medium-term persistence and variability.
Interaction terms: Multiplicative interactions such as rainfall × humidity and temperature × incidence were included to allow models to capture joint effects.
Seasonal indices: Month-of-year was encoded as a cyclical pair (sin, cos) to preserve continuity between December and January; categorical seasonal dummies were also prepared for models that accept categorical inputs.
Feature selection screening: Prior to final model training, pairwise correlations and mutual information scores were computed, and highly collinear variables were flagged. For tree-based models (LightGBM), full feature sets were initially used and later pruned by important metrics; for parametric models (GPR) a reduced subset was used to limit computational complexity.

All engineered features and preprocessing steps were saved as part of a pipeline object (scikit-learn Pipeline) to guarantee consistent treatment of train and test sets.

### 2.3 STL-based time series decomposition

To enhance interpretability and modeling robustness, Seasonal-Trend decomposition based on Loess (STL) (21) was applied to each time series. STL decomposes a time series *X_j_(t)* into three distinct components.


$$x_{j}(t)=T_{j}(t)+S_{j}(t)+R_{j}(t)$$

Where:

-
$T_{j}(t)$ : long-term trend component
-
$S_{j}(t)$ : seasonal component
-
$R_{j}(t)$ : residual (irregular or noise) component.

This decomposition enhances interpretability and aids in isolating underlying dynamics, crucial for robust forecasting. The decomposition relies on LOESS smoothing for trend and seasonality extraction. The residuals are calculated as:


$$R_{j}(t)=X_{j}(t)-(T_{j}(t)+S_{j}(t))$$

#### 2.3.1 STL configuration and rationale

- Seasonal period was fixed at 12 months to capture annual cycles. Robust LOESS options were enabled to mitigate influence of isolated anomalies.
- Smoothing windows for seasonal and trend components were chosen via sensitivity analysis with final settings selected to balance smoothness against fidelity to observed structure.
- STL was applied both to the target series (monthly mortality) and to key predictors (rainfall, humidity) so that hybrid models could be trained on decomposed components (trend, seasonal, residual) rather than on raw series only. This separation allows downstream models to learn different dynamics.

Decomposition improves interpretability (explicit seasonal and trend terms) and reduces burden on learning algorithms by isolating the irregular component where complex nonlinear dynamics are most evident (22, 23).

### 2.4 Multivariate time series modeling

The transformed and decomposed dataset was used to build predictive models (24). Let the multivariate dataset be defined as: $X=\{X_{1}(t),X_{2}(t),\ldots ,X_{p}(t)\},$ where $X_{j}(t)$ is the $j^{\mathrm{th}}$ time series at time *t*. and the complete data matrix is of size, p represents the quantity of time series, each encapsulating a distinct characteristic or measurement across a temporal span. The variable *t* specifies a discrete time index, spanning from *t =* 1 to *t* = *T*, with *T* being the total number of time steps in the collection. The dataset *X* constitutes a matrix of dimensions *t* by *p*, with each row representing a distinct time step and each column representing a separate time series. The objective of multivariate time series analysis is to describe the temporal dynamics, interactions, and dependencies among multiple time series to reveal patterns, infer causal linkages, or provide precise predictions (24, 25). The primary objective is to model the temporal dynamics and interdependencies among variables to accurately forecast future VL mortality.

To address the heterogeneous nature of the time series encompassing both periodic and non-periodic patterns, the following models were implemented:

- Gaussian Process Regression (GPR): for probabilistic, uncertainty-aware forecasting
- Long Short-Term Memory (LSTM): to capture long-range sequential dependencies
- Temporal Pattern Attention LSTM (TPA-LSTM): for attention-enhanced sequence learning, allowing the model to weigh informative time steps
- LightGBM: as a baseline tree-based gradient boosting method, effective for capturing non-linear relationships with high interpretability

This hybrid modeling pipeline was designed to produce robust and interpretable forecasts under variable epidemiological and climatic conditions, ultimately supporting timely public health responses in resource-limited, high-burden settings.

#### 2.4.1 The proposed hybrid STL-Gaussian process regression (GPR) model design

- For STL–GPR, we model the irregular component *R*_morality_*(t)* together with decomposed predictor residuals using Gaussian Process Regression (GPR) (26, 27). The predictive distribution enables explicit uncertainty quantification.
- Kernel choice: a composite kernel 𝑘 (·,·) formed by an RBF (squared exponential) kernel for smooth variations plus a WhiteKernel for observation noise:

$$k(x,x^{\prime })=\sigma_{f}^{2}\exp(-\frac{1}{2l^{2}}{\left\|x-x^{2}\right\|}^{2})+\sigma_{n}^{2}\delta_{x-x\prime }$$

Hyperparameters (length scale *ℓ*, signal variance $\sigma_{f}^{2}$ , noise variance $\sigma_{n}^{2}$ ) were estimated by maximizing the marginal likelihood.

- Computational considerations: GPR scales as *O*(𝑛^3^) with training size 𝑛 given the moderate sample size (monthly data, T ≈ 180), exact GPR was tractable. For larger datasets, sparse approximations (e.g., inducing points) would be considered.

##### 2.4.1.1 Uncertainty

The posterior predictive distribution yields mean forecasts and credible intervals (95% prediction intervals) used in downstream evaluation.

#### 2.4.2 The proposed hybrid STL-long short-term memory (LSTM) model

The STL–LSTM model combines STL decomposition with Long Short-Term Memory (LSTM) networks to model non-linear temporal dependencies. After decomposition, the seasonal component is removed, and the trend and residual signals—which contain both short-term noise and long-term dependencies—are used as input to the LSTM model (28, 29).

LSTM is a specialized form of recurrent neural network (RNN) that overcomes the vanishing gradient problem through its gated architecture (input, forget, and output gates). This enables it to retain memory across long sequences, making it ideal for epidemiological time series where climatic drivers and infection cycles can influence outcomes with significant time lags.

By applying LSTM to the decomposed components, the model learns to associate delayed climatic variations (e.g., temperature, rainfall) with future mortality patterns. This hybrid structure enhances predictive accuracy compared to applying LSTM directly to raw, noisy data.

##### 2.4.2.1 Model design

- The inputs comprised aggregated feature vectors created by concatenating the STL components of trend, seasonality, and residuals alongside lagged epidemiological and climatic predictors (*t* − 1, *t* − 2, *t* − 3).
- In this context, “concatenation” denotes the amalgamation of various components into a singular multivariate input vector utilized for model training. The sliding window of residuals represents sequential residual observations used to capture short-term temporal dependencies in the series.
- Architecture: stacked LSTM with two recurrent layers. Typical hyperparameters used (baseline) were: 2 layers × 64 units, dropout 0.2 after each LSTM layer, dense output layer with linear activation.
- Loss and optimization: mean squared error (MSE) loss, optimized with Adam (initial learning rate $1*10^{-3}$ ).
- Training: mini-batch training (batch size 16), early stopping monitoring validation loss with patience 20 epochs, maximum 200 epochs.
- LSTM processes decomposed signals (trend + residuals) by updating hidden states using gated mechanisms. For each time step 𝑡:

$$f_{t}=\sigma (W_{f}[h_{t-1},x_{t}]+b_{f})$$

$$i_{t}=\sigma (W_{f}[h_{t-1},x_{t}]+b_{i})$$

$${\tilde{C}}_{t}=\tan h(W_{c}[h_{t-1},x_{t}]+b_{c})$$

$$C_{t}=(f_{t}⊙C_{t-1},i_{t}⊙{\tilde{C}}_{t})$$

$$o_{t}=\sigma (W_{o}[h_{t-1},x_{t}]+b_{o})$$

$$h_{t}=o_{t}⊙\tan h(C_{t})$$

- Here, *f_t,_ i_t_,* and *o_t_* are forget, input, and output gates, respectively. *C_t_* is the cell state, and *h_t_* is the hidden state. This allows the STL–LSTM to retain long-term dependencies from decomposed temporal features.

##### 2.4.2.2 Rationale

LSTM networks are adept at modeling long-range temporal dependencies and are therefore suitable for learning lagged climatic influences that affect mortality with delays.

#### 2.4.3 The proposed hybrid STL-temporal pattern attention with LSTM (STL-TPA-LSTM)

The STL–TPA-LSTM model extends the STL–LSTM approach by incorporating a Temporal Pattern Attention (TPA) mechanism. While STL–LSTM can model sequential dependencies, it assigns equal importance to all past time steps. TPA overcomes this limitation by learning to dynamically attend to the most relevant temporal segments across lagged features (30).

After STL decomposition, the trend and residual signals are processed by the LSTM, followed by an attention layer. The attention weights allow the model to focus on critical historical events, such as sudden climatic anomalies or infection surges, which disproportionately affect future mortality.

This attention-based hybrid design not only improves forecasting accuracy but also enhances interpretability, since the attention weights can be analyzed to understand which past events most strongly influenced the predictions. Such interpretability is particularly important in epidemiology, where domain experts need to trace causal pathways between climate factors and disease dynamics.

##### 2.4.3.1 Model design

- This architecture augments the LSTM backbone with a temporal attention mechanism that computes context-aware weights over past time steps. Let the LSTM produce hidden states $\left\{h_{t-r}\right\}$ for a sliding window; attention weights $\alpha_{\tau }$ ​ are computed as:

$$e^{\tau }=v^{⊤}\tan h(W_{h}h_{t-\tau }+b),\alpha_{\tau }=\frac{\exp(e_{\tau })}{\sum_{k}\exp(e_{k})}$$

and the context vector $c_{\tau }=\sum_{t}\alpha_{t}h_{t-\tau }$ is concatenated with the current LSTM output to produce the final prediction.

- Architecture and training hyperparameters mirrored the STL–LSTM baseline, with an additional attention projection layer (size 32).

##### 2.4.3.2 Advantages

- Attention enables the model to emphasize temporally informative segments (for example, anomalous rainfall months) and thus can better capture episodic drivers of mortality.

#### 2.4.4 The proposed hybrid STL-light gradient boosting machine (LightGBM) model

The STL–LightGBM model integrates STL decomposition with Light Gradient Boosting Machine (LightGBM), a decision-tree-based ensemble learning method. Following decomposition, the trend, seasonal, and residual components are used as engineered features for LightGBM training (31).

LightGBM builds an ensemble of decision trees using gradient boosting, optimizing prediction by sequentially correcting residual errors. It is computationally efficient, supports missing values, and provides feature importance rankings, making it suitable for high-dimensional tabular datasets derived from epidemiological and climatic indicators.

This hybrid approach is particularly effective in settings where the relationships between predictors and outcomes are highly non-linear and interaction-driven. Compared to deep learning models, LightGBM offers faster training while still achieving competitive predictive accuracy. Its ability to highlight key predictive features (e.g., rainfall or temperature indicators) further supports interpretability in public health applications.

##### 2.4.4.1 Model design

- Inputs: engineered features including STL components (trend, seasonal, residual) and lagged and interaction predictors, presented as tabular features to LightGBM.
- Baseline hyperparameters: objective = *regression*, metric = *rmse*, *num_leaves* = 31, *learning_rate* = 0.05, *n_estimators* up to 1,000 with early stopping (50 rounds) on a validation set.
- Feature importance and SHAP values were used to interpret the model and to rank the contribution of each input (STL components vs. raw predictors).

##### 2.4.4.2 Rationale

LightGBM is an efficient gradient-boosted tree method that handles nonlinear interactions and missing values well and typically provides strong performance on tabular datasets derived from decomposed time series.

##### 2.4.4.3 Hybrid strategy summary

For all hybrid variants we explored two composition strategies:

1. Direct modeling of components: train a model to predict *R*_mortality_(*t*) and then reconstruct the full forecast as $\hat{X}(T)=\hat{T}(t)+\hat{R}(t)$ . Trend and seasonal components were optionally extrapolated using simple extrapolation (e.g., linear trend continuation) or left as STL-extracted values depending on model.
2. Joint modeling of components and predictors: concatenate component vectors from multiple series (mortality residuals, rainfall residuals, etc.) and train models jointly on the richer representation.

### 2.5 Model evaluation and pseudocode

To prevent information leakage and preserve temporal dependence, a chronological division was implemented. Model training was conducted using data from January 2002 to December 2018, while out-of-sample testing was confined to data from January 2019 to December 2022. A rolling-origin time-series validation technique was employed during model building to assess model stability across multiple time segments. Multiple statistical indices were employed to ensure a balanced evaluation of model accuracy, error magnitude, and explanatory power.

- Root Mean Squared Error (RMSE): Penalizes large deviations between predicted and observed values (32).

$$\text{RMSE}=\sqrt{\frac{1}{n}\sum_{i=1}^{n}{\left(y_{i}-{\hat{y}}_{i}\right)}^{2}}$$

- Mean Absolute Error (MAE): Provides the average magnitude of prediction errors, making it more robust to outliers than RMSE (32).

$$\mathrm{MAE}=\frac{1}{n}\sum_{i=1}^{n}|y_{i}-{\hat{y}}_{i}|$$

- Coefficient of Determination (*R*^2^): Quantifies how much of the variance in the observed data is explained by the model (33).

$$R^{2}=1-\frac{\sum_{i-1}^{n}{\left(y_{i}-{\hat{y}}_{i}\right)}^{2}}{\sum_{i-1}^{n}{\left(y_{i}-{\bar{y}}_{i}\right)}^{2}}$$

- Mean Absolute Percentage Error (MAPE): Expresses errors as percentages of observed values, allowing for scale-independent comparison (33).
- Willmott’s Index of Agreement (*d*): Measures the degree of model prediction error relative to observed deviations from the mean, with values closer to 1 indicating higher agreement.
- Percent Bias (PBIAS): Detects systematic over- or under-prediction, with values near zero denoting unbiased performance.

Together, these metrics provide a comprehensive and multi-dimensional assessment of model predictive capabilities, essential for validating hybrid forecasting methods in public health applications.

#### 2.5.1 Pseudocode

# **Input**: raw monthly series: mortality,incidence,rainfall,temp,humidity
# **Output**: forecasted mortality series + prediction intervals
# **1. Preprocessing**data = load_data()
data = align_monthly(data)
data = impute_missing(data,method=′linear + kalman′)
data = generate_lag_features(data,lags=[1,2,3])
data = generate_rolling_stats(data,windows=[3,6])
data = create_interactions(data,[′rainfall′,′humidity′],[′temp′,′incidence′])
data_scaled = minmax_scale(data)
# **2. STL decomposition**components = {}
for series in [′mortality′,′rainfall′,′humidity′,′temp′]:
 T,S,R = stl_decompose(data_scaled[series],period = 12,robust = True)
 components[series] = {′trend′:T,′seasonal′:S,′residual′:R}
# **3. Prepare model inputs**X_train,X_test,y_train,y_test = time_train_test_split(components,train_size=0.8)
# **4. Train models**models = {}
# GPR
models[′gpr′] = train_gpr(X_train[′residuals′],y_train[′residuals′],kernel=′RBF+White′)
# LSTM
models[′lstm′] = train_lstm(X_train[′seq_inputs′],y_train,params={units:64,layers:2,dropout:0.2})
# TPA-LSTM
models[′tpa_lstm′] = train_tpa_lstm(X_train[′seq_inputs′],y_train,attention=True,params=...)
# LightGBM
models[′lgb′] = train_lightgbm(X_train[′tabular′],y_train,params={num_leaves:31,lr:0.05})
# **5. Forecast and evaluate**for name,model in models.items():
 y_pred,y_pi = model.predict(X_test,return_interval=True)
 metrics = evaluate(y_test,y_pred,measures=[′MAE′,′RMSE′,′MAPE′,′R2′,′Willmott′,′PBIAS′])
 print(name,metrics)


### 2.6 Implementation

All experiments were conducted in Python (v3.10) to ensure reproducibility and interoperability with widely used machine learning frameworks. The following libraries were employed in the pipeline:

- Statsmodels: for Seasonal and Trend decomposition using LOESS (STL) to separate trend, seasonality, and residual components of the time series.
- Scikit-learn: for implementing Gaussian Process Regression (GPR), hyperparameter optimization, and evaluation metrics (RMSE, MAE, MAPE, *R*^2^, etc.).
- TensorFlow/Keras: for constructing and training LSTM and TPA-LSTM models, with GPU acceleration to handle computationally intensive training.
- LightGBM: for implementing the gradient boosting framework, allowing efficient handling of structured tabular data derived from decomposed features.

To ensure scalability and reproducibility, random seeds were fixed, preprocessing steps were standardized, and hyperparameters were tuned using grid search and cross-validation. All models were trained on a workstation equipped with an NVIDIA GPU and 32 GB RAM, providing sufficient computational capacity for deep learning–based experiments.

The modular design of the pipeline ensures that the same framework can be extended to real-time deployment in public health monitoring systems. This makes it suitable not only for retrospective mortality forecasting but also for prospective early-warning applications in epidemiological surveillance.

## 3 Results and discussion

This section presents the outcomes of applying the four proposed hybrid models—STL-GPR, STL-LSTM, STL-TPA-LSTM, and STL-LightGBM—for forecasting visceral leishmaniasis (VL) mortality in Gedaref, Sudan. Performance is evaluated using MAE, RMSE, MAPE, *R*^2^, the Willmott Index, and PBIAS. In addition to numerical results, we offer comprehensive insights into how climate variability influences VL mortality.

### 3.1 Descriptive insights from data

**Figure 2:**
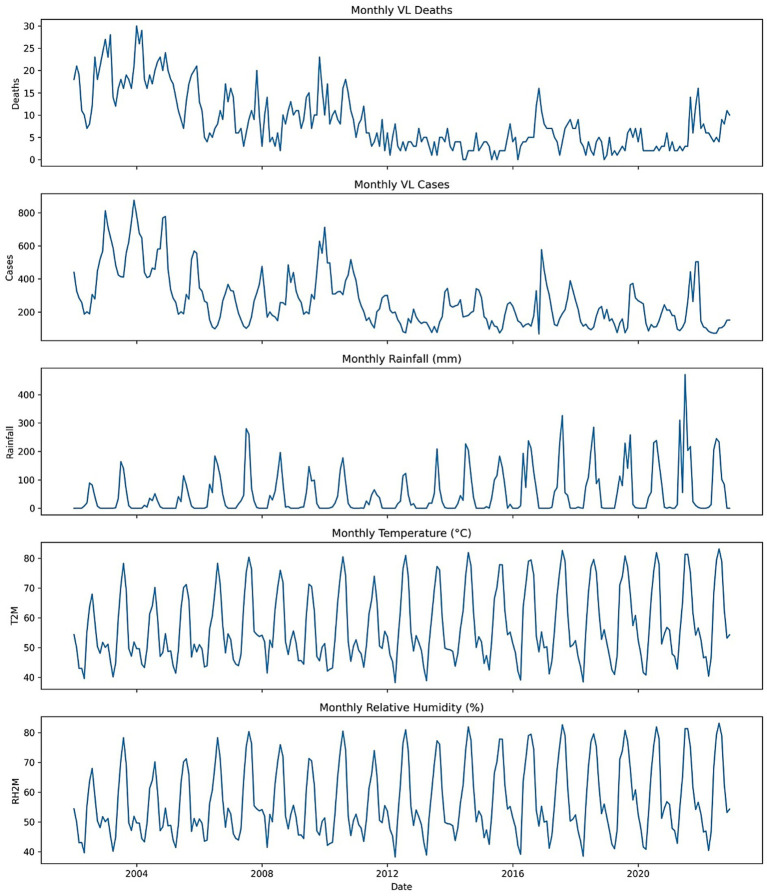
Monthly time series of visceral leishmaniasis (VL) deaths, cases, rainfall, temperature, and relative humidity in Sudan (2002–2022).

Figure 2 shows the time series on mortality, case numbers, precipitation, temperature and relative humidity. VL deaths have a marked seasonality, with repeated peaks during the rainy seasons and with both rainfall and humidity exhibiting substantial year-on-year variability. There is an inverse relationship between mortality probability and temperature, which makes such a curve quite predictable.

**Figure 3:**
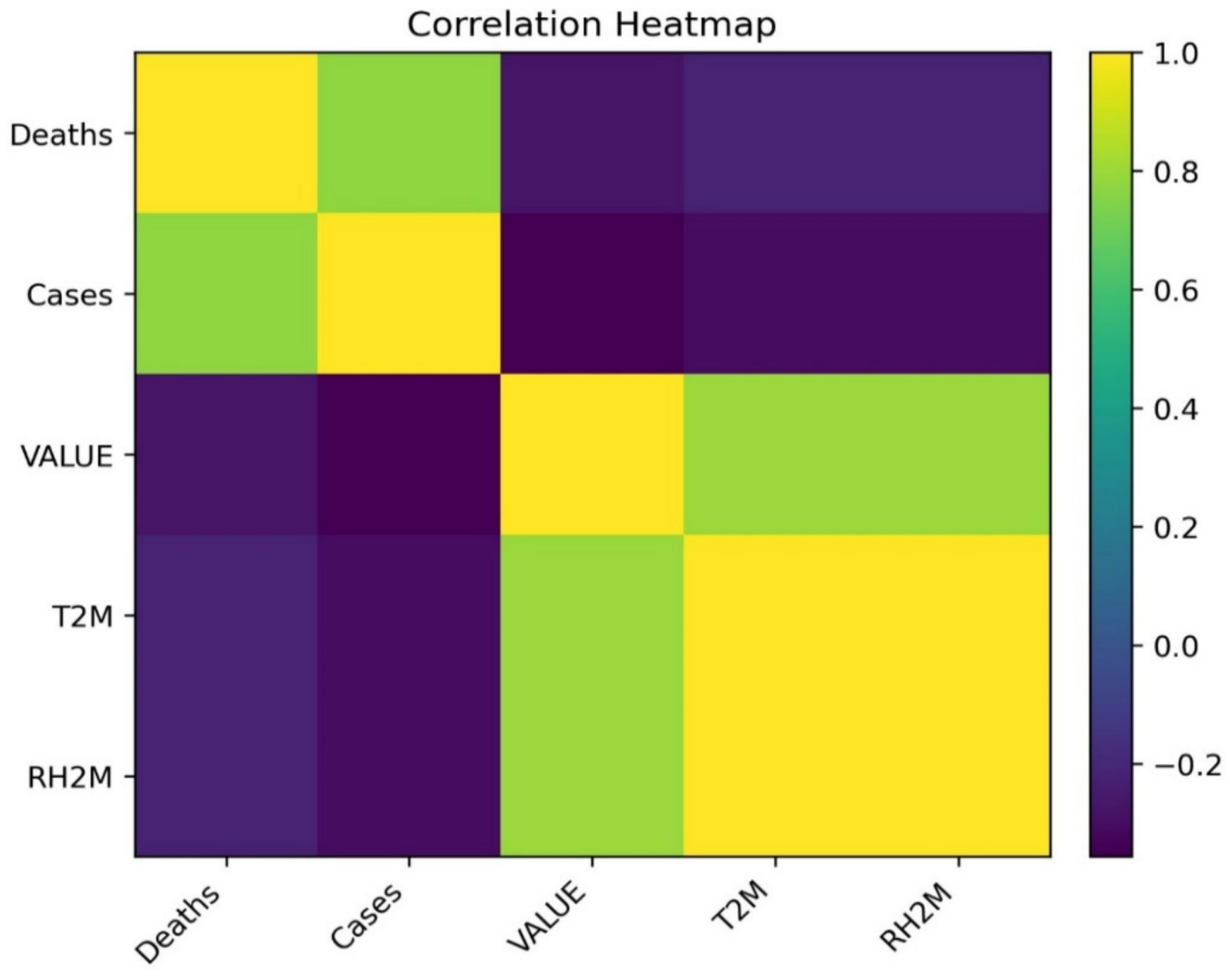
Correlation heatmap showing relationships among VL deaths, cases, and climatic variables.

**Figure 4:**
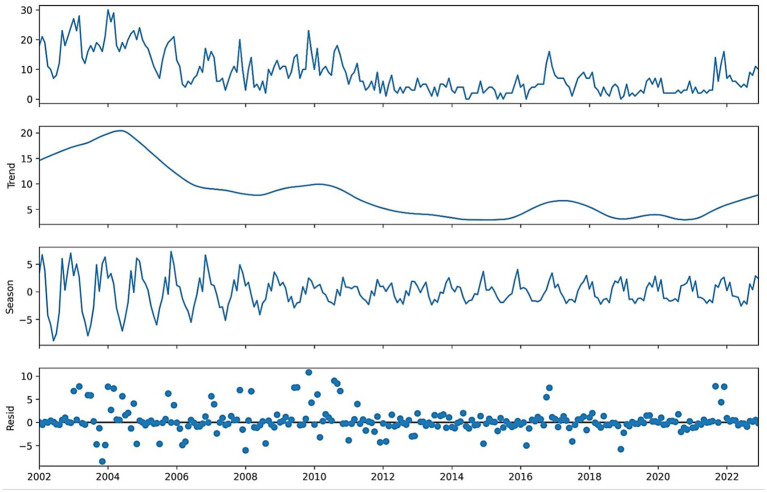
STL decomposition of monthly VL deaths into trend, seasonal, and residual components.

**Figure 5:**
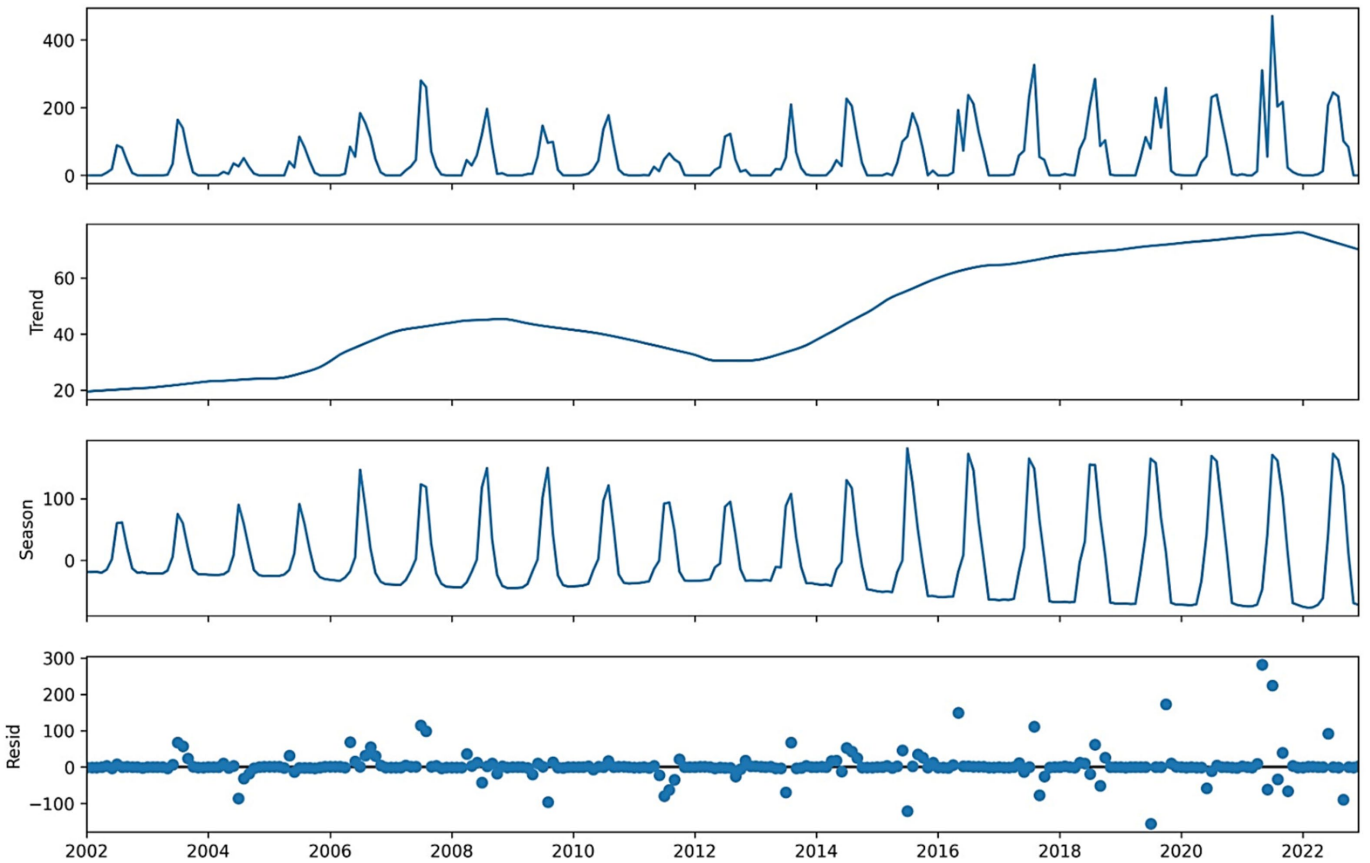
STL decomposition of monthly rainfall into trend, seasonal, and residual components.

The correlation heatmap (Figure 3) confirms these relationships, showing strong positive correlations between deaths and cases, and notable associations with rainfall and humidity. The STL decomposition also uncovers structural patterns. Mortality decomposition (Figure 4) shows consistent long-term declining patterns since 2002, characterized by pronounced seasonality, while rainfall decomposition (Figure 5) reveals enduring yearly cycles. Together, these insights underscore the epidemiological relevance of climate variability and the appropriateness of STL preprocessing.

The supplementary ACFs and PACFs (Supplementary Figures S1–S4) demonstrate the presence of temporal dependence and indicate the necessity of including a lag of climatic predictors in forecasting models.

### 3.2 STL-LightGBM performance

**Figure 6:**
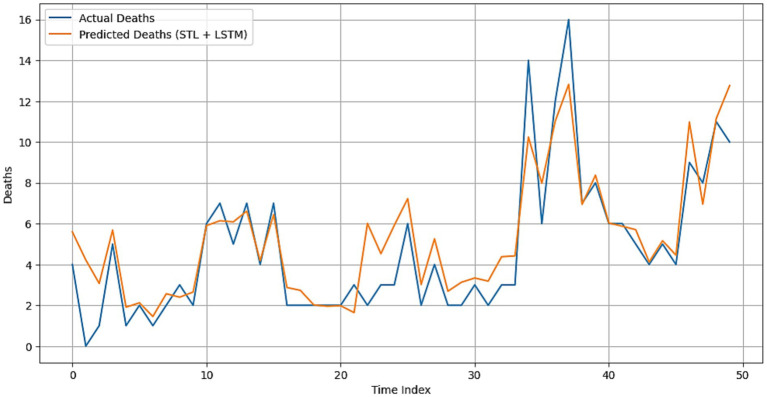
Actual versus predicted VL deaths using the hybrid STL + LSTM model.

STL-LightGBM experienced better prediction efficacy compared to all other models. The model achieved a Mean Absolute Error (MAE) of 0.5410, a Root Mean Square Error (RMSE) of 0.7650, and a Mean Absolute Percentage Error (MAPE) of 15.43%, with a coefficient of determination (*R*^2^) of 0.9491. The Willmott Index ultimately yielded a value of 0.9861, while PBIAS approached zero at 0.33%. As seen in Figure 6, it closely aligns with the measured mortality curve by accurately reflecting its peaks and troughs. These results confirm the ability of LightGBM to exploit nonlinear relationships and interactions in multivariate climate–disease data.

The performance of the STL-LightGBM model confirms its ability to explain the complex nonlinear interactions between climatic and epidemiological variables. The importance of humidity patterns and delayed infection indicates that the mortality rate of visceral leishmaniasis is affected by environmental conditions favorable to sandfly reproduction and delayed infection cycles. The results align with the environmental conditions of vectors in eastern Sudan, where vector populations and parasite development are influenced by factors such as humidity and precipitation.

From a public health perspective, the model’s accuracy demonstrates its potential use in early warning systems. By forecasting climatic and epidemiological factors before to peak mortality, the model facilitates proactive measures including vector control, improved surveillance, and better resource allocation.

### 3.3 STL-TPA-LSTM performance

**Figure 7:**
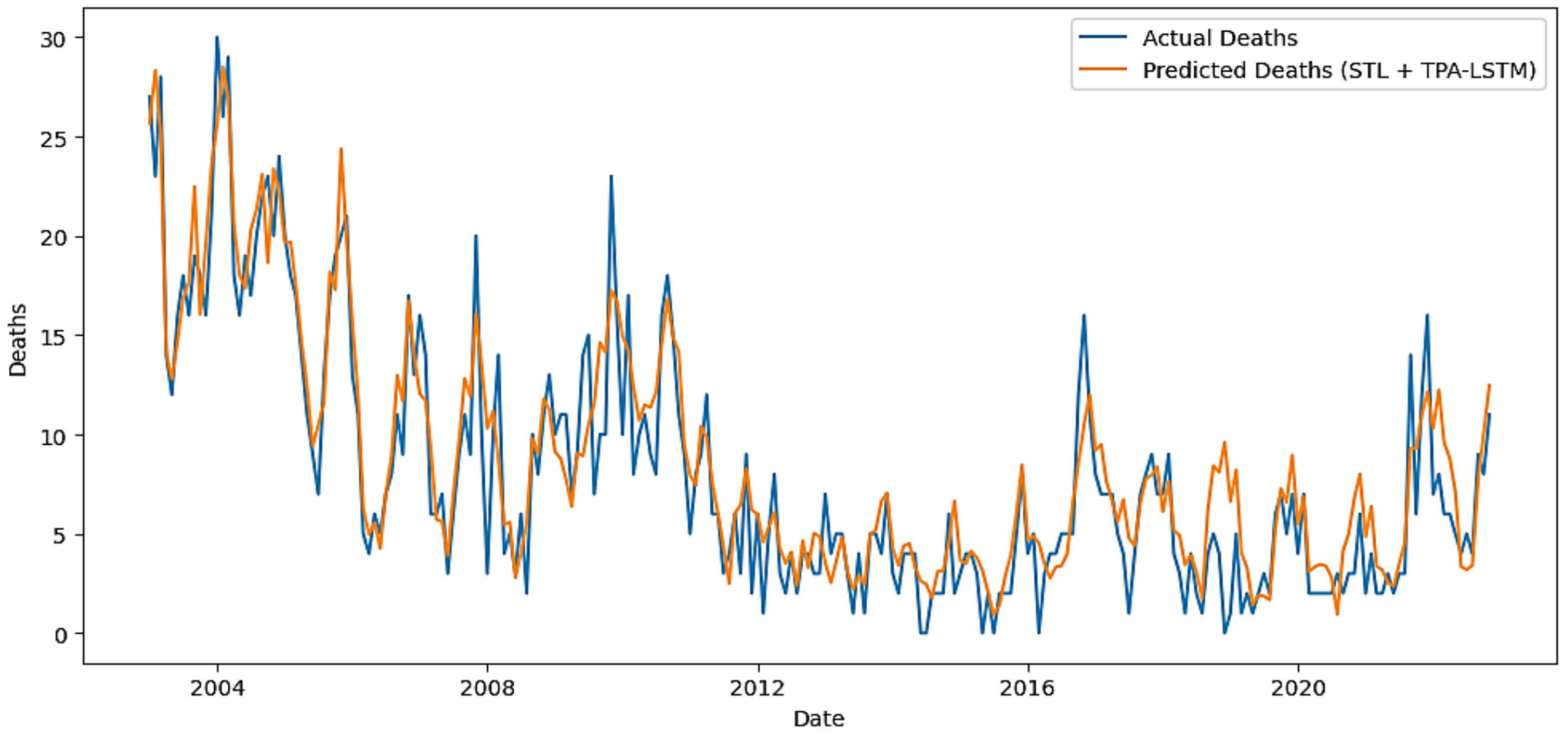
Actual versus predicted VL deaths using the hybrid STL + TPA-LSTM model.

The STL-TPA-LSTM model ranked second, benefiting from the temporal attention mechanism. It recorded MAE = 0.92, RMSE = 1.32, MAPE = 25.00%, and *R*^2^ = 0.85. The Willmott Index was 0.96 and PBIAS 0.80%. As shown in Figure 7, the model demonstrates stronger responsiveness to local variations than standard LSTM, highlighting the contribution of attention in emphasizing influential temporal segments.

The enhanced efficacy of STL-TPA-LSTM relative to conventional LSTM underscores the significance of temporally localized events in VL dynamics. Attention processes enabled the model to emphasize significant climatic anomalies and outbreak-related indicators, indicating that VL mortality is influenced by sporadic ecological disturbances rather than solely by consistent seasonal patterns.

This underscores the epidemiological insight that abrupt environmental alterations—such as increases in rainfall or humidity—can precipitate transmission escalation. Such findings are crucial for developing adaptive monitoring systems that respond to environmental cues rather than relying solely on historical averages.

### 3.4 STL-LSTM performance

**Figure 8:**
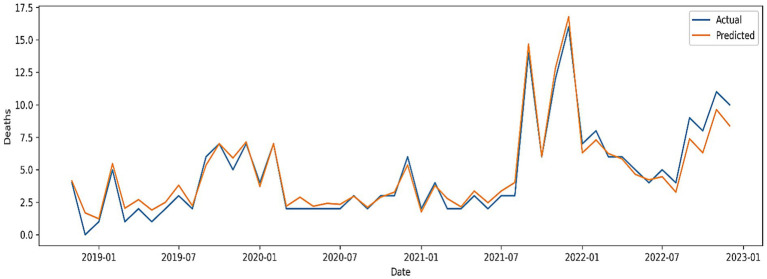
Actual versus predicted VL deaths using the hybrid STL + LightGBM model.

The STL-LSTM model achieved reasonable performance with MAE = 0.97, RMSE = 1.39, and MAPE = 31.88%. It yielded a *R*^2^ of 0.83 and a Willmott Index of 0.95; nevertheless, it struggled to fully capture sudden shifts. As seen in Figure 8, akin to the overall mortality, our model performs well for the long-term trend of influenza but has limited predictive capability for abrupt peaks and declines.

The STL-LSTM model effectively identified long-term temporal connections; however, it had restricted capacity to capture abrupt increases in mortality. The mortality of visceral leishmaniasis is affected not only by progressive climate trends but also by abrupt environmental and epidemiological changes. This tendency aligns with the transmission dynamics stemming from illness outbreaks.

From a health planning standpoint, these findings indicate that this model, which depends exclusively on sequential memory, may undervalue abrupt increases in mortality, underscoring the necessity of hybrid models that incorporate both linear and nonlinear interactions within predictive monitoring systems.

### 3.5 STL-GPR performance

**Figure 9:**
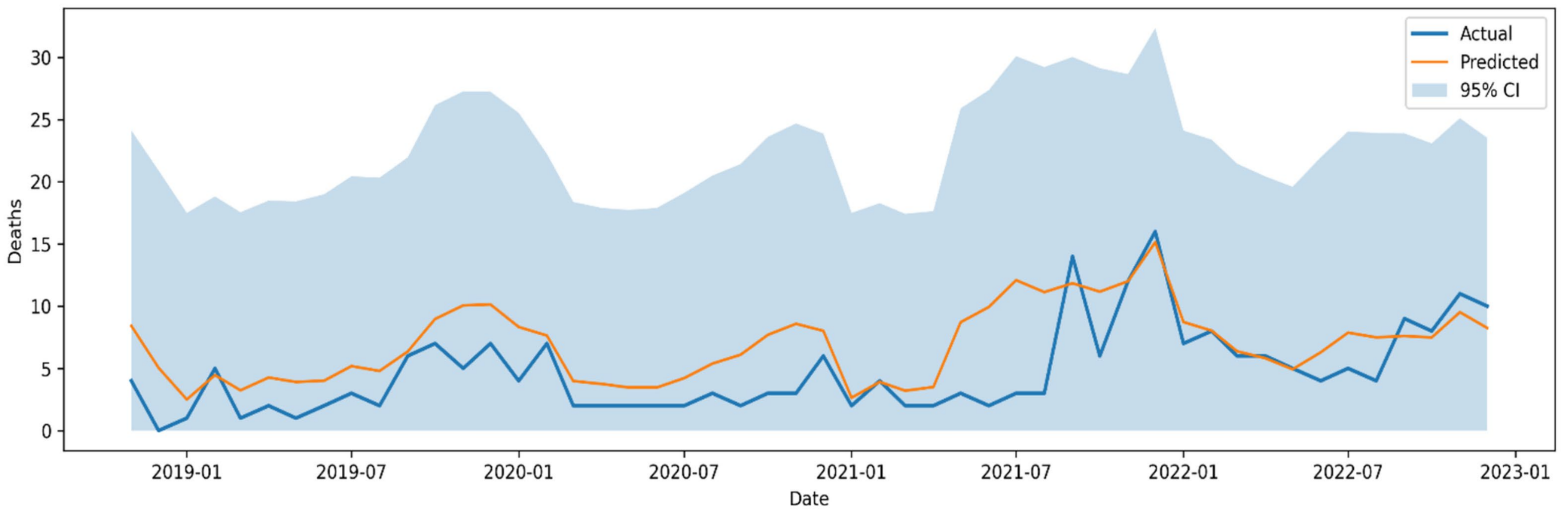
Actual versus predicted VL deaths using the hybrid STL + GPR model with 95% confidence intervals.

While STL-GPR demonstrated the lowest accuracy overall, it offered significant insights into uncertainty quantification. The model produced MAE = 1.08, RMSE = 1.51, and MAPE = 39.75%, with *R*^2^ = 0.81, Willmott = 0.94, and PBIAS = 11.84%. Figure 9 illustrates the predictive curve with 95% confidence intervals, showing its capacity to approximate general mortality dynamics but with reduced sharpness in capturing extremes.

Interestingly, the stability of STL-GPR was enhanced when combined with PCA dimensionality reduction, while predictive accuracy decreased (MAE = 2.36, RMSE = 2.68, *R*^2^ = 0.39). This trade-off underscores the sensitivity of nonparametric Bayesian models to the scaling of features and the dimensionality involved.

While the STL-GPR model attained satisfactory forecast accuracy, it offers significant probabilistic insights into the uncertainty associated with monthly mortality statistics. This discovery is especially significant in resource-constrained environments. The capacity to create intervals of uncertainty facilitates risk-informed decision-making and aids public health authorities in preparing for worst-case mortality scenarios.

**Figure 10:**
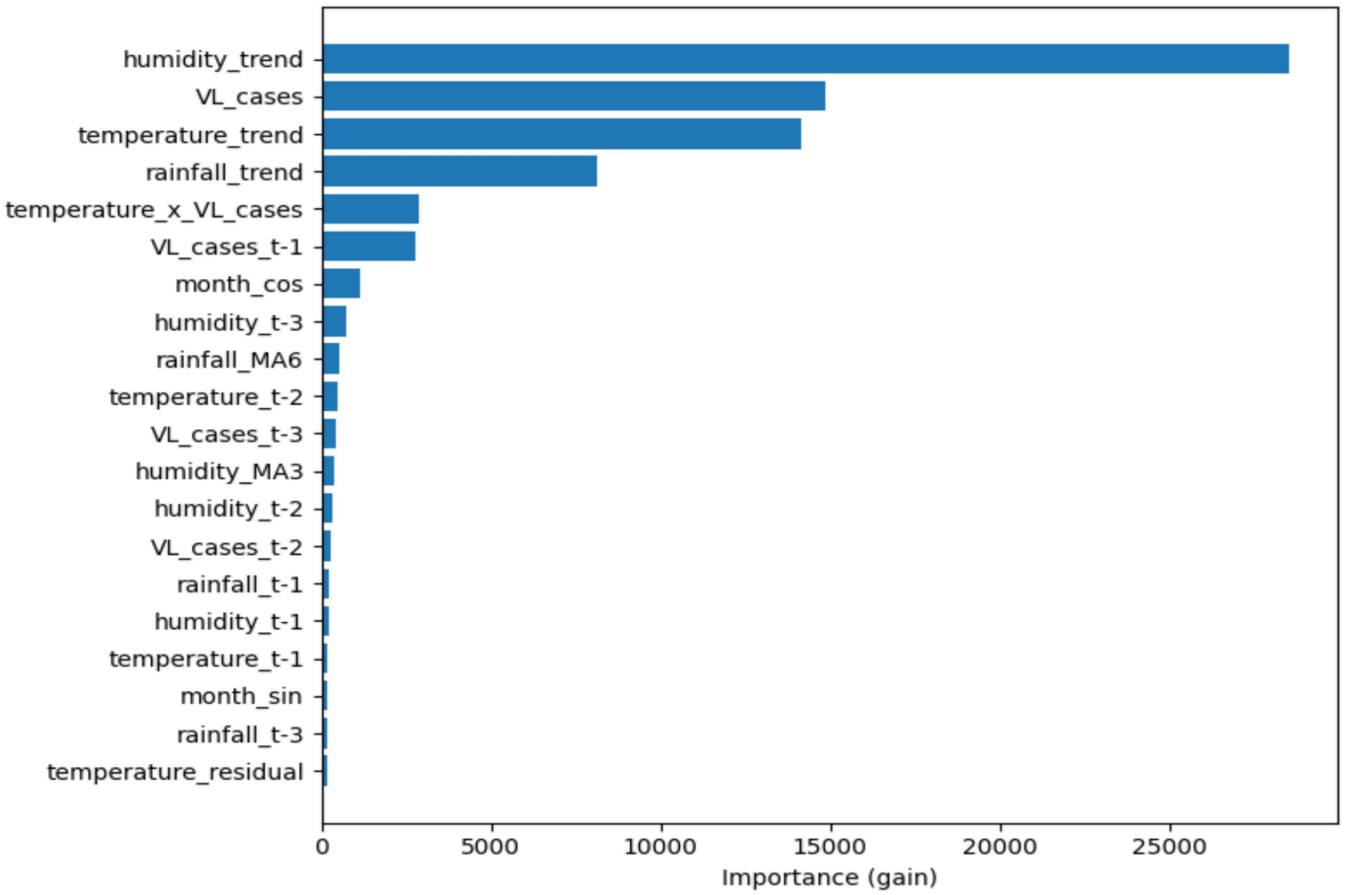
Feature importance and SHAP-based interpretation of predictors driving VL mortality forecasts using the STL-LightGBM model.

Figure 10 illustrates the feature importance analysis derived from the STL-LightGBM model, indicating that humidity trends, VL incidence, and temperature-related variables are the predominant predictors of VL mortality. SHAP interpretation further substantiates these findings, emphasizing the predominant influence of environmental trends and delayed epidemiological signals (t-1, t-2, t-3) in formulating mortality predictions and enhancing model interpretability within the multivariate forecasting paradigm.

### 3.6 Comparative insights

**Figure 11:**
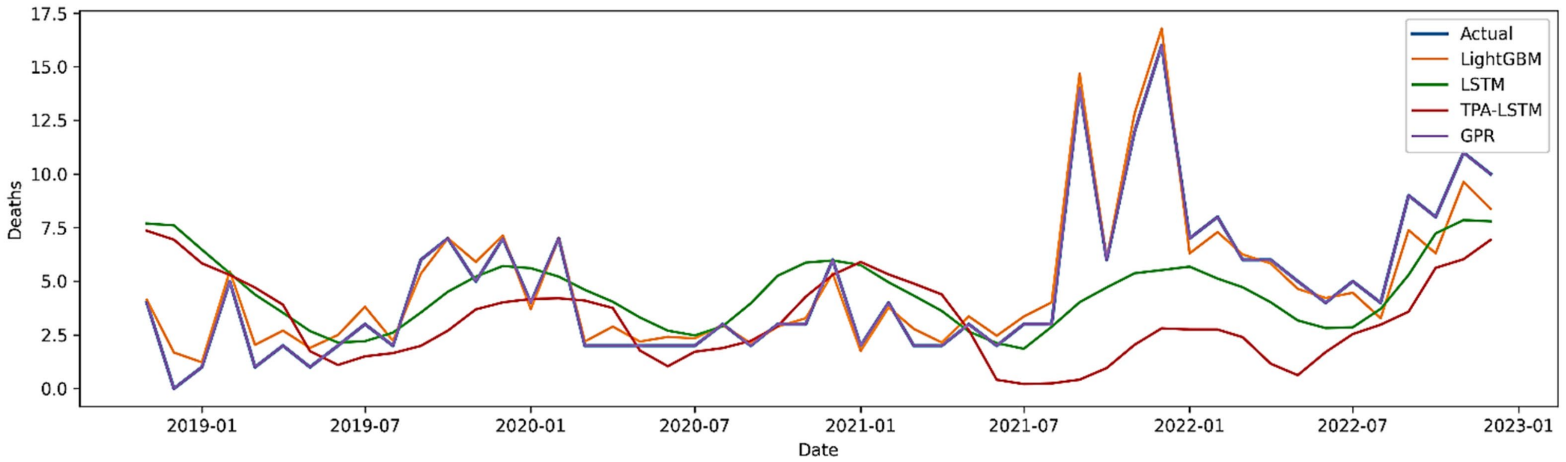
Comparative performance of hybrid models (STL-GPR, STL-LSTM, STL-TPA-LSTM, STL-LightGBM) for VL mortality forecasting.

The comparative plot (Figure 11) and Table 3 shows both the predictions of all models and the reported mortality. STL-LightGBM is the best in both accuracy and robustness, while STL-TPA-LSTM and STL-LSTM are also comparable. Despite its lower degree of accuracy, STL-GPR remains useful for generating probabilistic predictions that help make decisions when facing uncertainty, even though working with little or no data.

**Table 3:** Comparative performance metrics of hybrid STL-based models for VL mortality forecasting.

Model	MAE	RMSE	MAPE (%)	*R* ^2^	Willmott	PBIAS (%)
STL + LightGBM	0.5410	0.7650	15.43	0.9491	0.9861	0.33
STL + TPA-LSTM	0.9200	1.3200	25.00	0.8500	0.9600	0.80
STL + LSTM	0.9700	1.3900	31.88	0.8300	0.9500	−5.22
STL + GPR	1.0800	1.5100	39.75	0.8100	0.9400	11.84

These results correspond with current research using STL decomposition alongside machine learning and deep learning, reinforcing the significance of hybrid architectures in enhancing seasonal illness predictions.

Comparative analyses indicate that models incorporating nonlinear interactions and environmental variables surpass conventional time-series and probabilistic models. They assert that the mortality rate of visceral leishmaniasis in Al-Qadarif state is affected by a confluence of delayed epidemic transmission, climate fluctuations, and environmental reactions to vector populations.

The findings emphasize that forecasting mortality rates, rather than only infection rates, offers valuable insights for healthcare systems, as peak mortality indicates deficiencies in early identification, treatment delivery, and environmental management. Consequently, predictive models centered on mortality rates can directly aid in prioritizing interventions and allocating resources in endemic regions.

## 4 Conclusion

This work presents and substantiates a comprehensive hybrid methodology for predicting mortality from visceral leishmaniasis using multivariate climate-sensitive time series data. The combination of STL decomposition with machine learning and deep learning models significantly enhanced the predictive accuracy of all the models applied. The STL-LightGBM model demonstrated excellent performance under all criteria (MAE, RMSE, MAPE, and *R*^2^), which was indicative of its ability to capture the non-linearities, the long-term dependencies and the seasonal patterns.

These models, when operationalised, have yielded a great many critical outcomes. STL decomposition is an effective preprocessing method that significantly enhances the model’s interpretability and accuracy in complex epidemiological series.

- The role of environmental factors, temperature, rainfall, and humidity, is key to predicting mortality from VL.
- LightGBM has strong advantages in terms of performance when combined with STL on both datasets due to the innate capability of LightGBM to process nonlinear and high-dimensional interactions well.
- Although the deep learning methods (LSTM and TPA-LSTM) had competitive performance, their optimal performance might only be achieved on larger datasets and with more extensive optimization, to outperform LightGBM on the same task.

The models in this article provide good decision-making support for public health authorities in endemic areas to target planning of interventions and allocation of resources.

## 5 Limitations

This study possesses multiple shortcomings that warrant acknowledgment.

First, the analysis relied on routine surveillance data collected by the Visceral Leishmaniasis Control Program of the Ministry of Health in Al Qadarif State. This data may be underreported, particularly in rural and resource-limited areas.

Second, the analysis focused on monthly visceral leishmaniasis mortality figures, as the primary objective of this study was to predict multivariable time series data for temporal dynamics and early warning signals, not to estimate epidemic risk. Furthermore, long-term monthly population data for Al Qadarif State were unavailable throughout the study period, limiting the ability to construct reliable mortality rates.

Third, Although significant meteorological influences were incorporated, additional environmental and structural elements were also considered such as vector control interventions, land-use changes, migration patterns, and access to healthcare were not explicitly included due to data limitations.

Fourth, the modeling framework was designed for short- to medium-term operational forecasts, not for long-term climate change projections. Therefore, the results should be interpreted as early warning indicators, not as simulations of long-term scenarios.

Fifth, although hybrid machine learning models have demonstrated strong predictive performance, causal inference remains limited, and the models should be interpreted as predictive tools rather than automated representations of the transmission dynamics of visceral leishmaniasis.

Finally, despite the application of time-based validation, external validation using independent datasets from other endemic areas in East Africa would enhance the generalizability and robustness of the proposed framework.

## Data Availability

The raw data supporting the conclusions of this article will be made available by the authors, without undue reservation.
