# Supplementary material for: Early-warning prediction of visceral leishmaniasis mortality using a multivariate STL–deep learning hybrid approach on 20 years of monthly time series
Source: Front Public Health. 2026 Mar 26;14:1754966. doi: 10.3389/fpubh.2026.1754966 (PMC13062275; doi:10.3389/fpubh.2026.1754966)
Supplement: Supplementary file 1 [file Supplementary_file_1.docx]

Supplementary Material

# Supplementary Figures


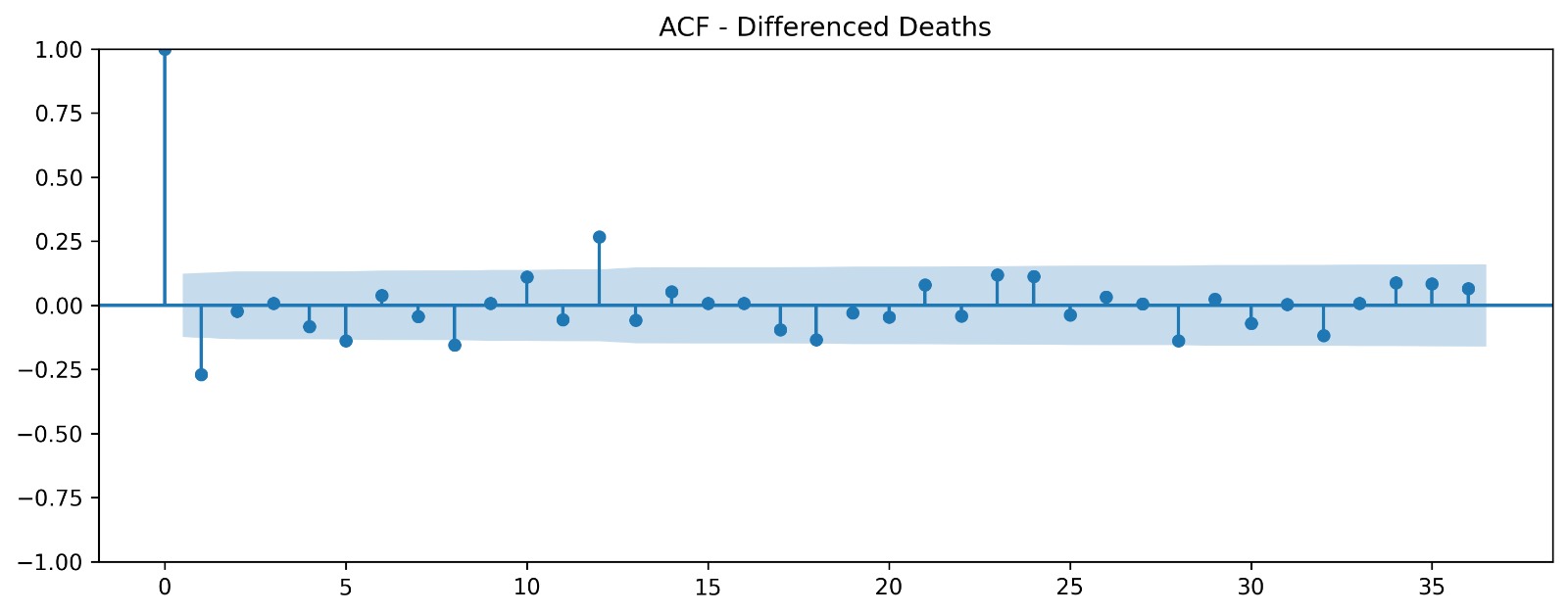


**Supplementary Figure 1.** Autocorrelation Function (ACF) of differenced VL deaths


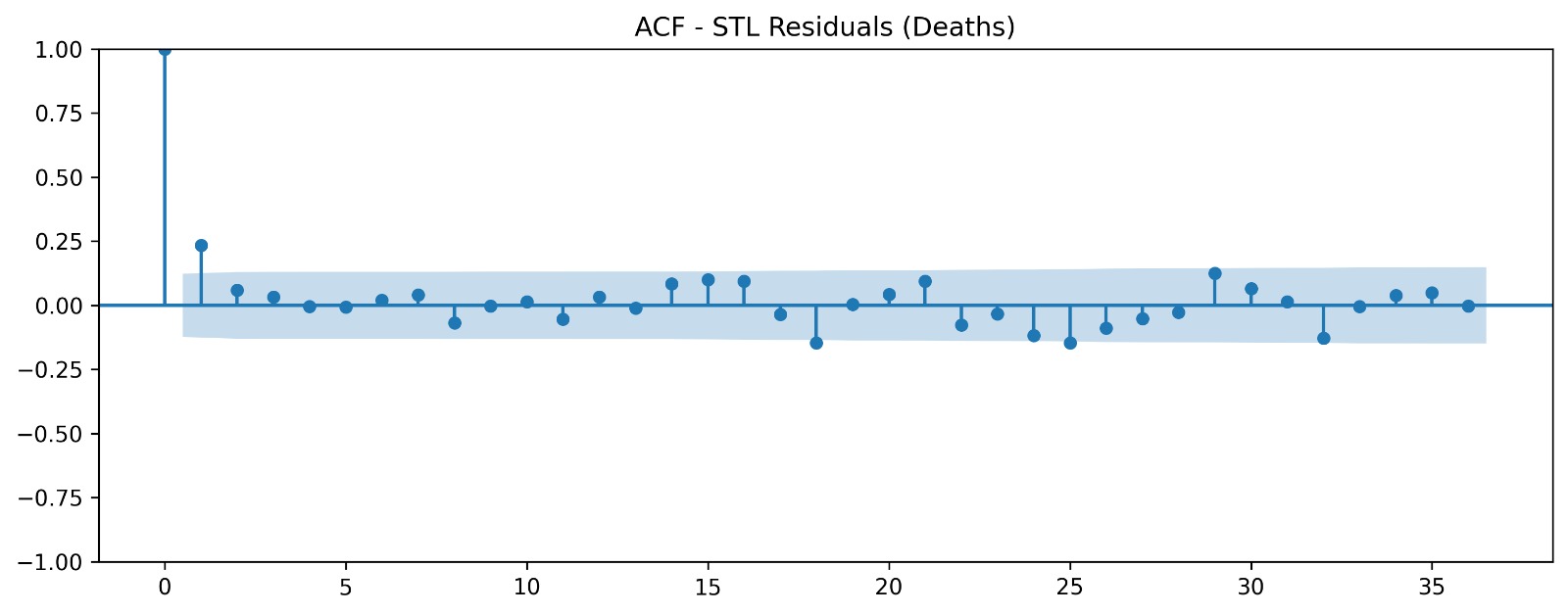


**Supplementary Figure 2.** ACF of STL residuals (VL deaths)


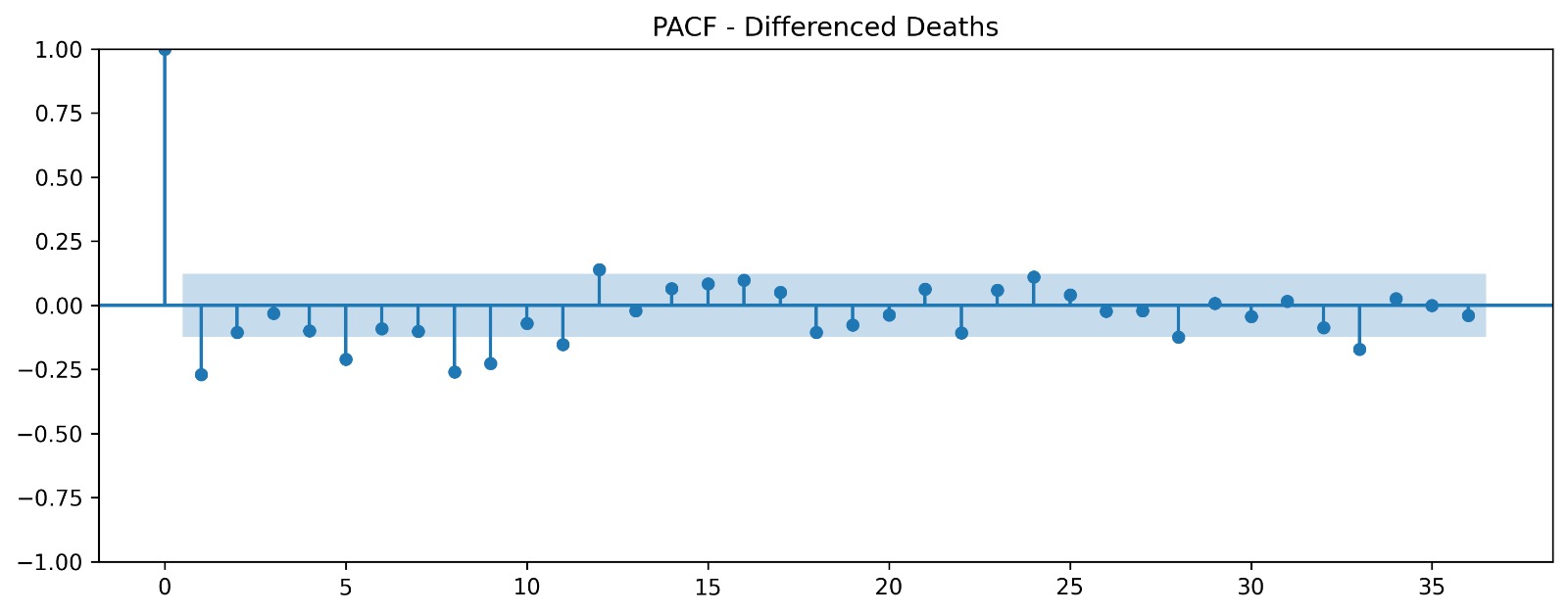


**Supplementary Figure 3.** Partial Autocorrelation Function (PACF) of differenced VL deaths.


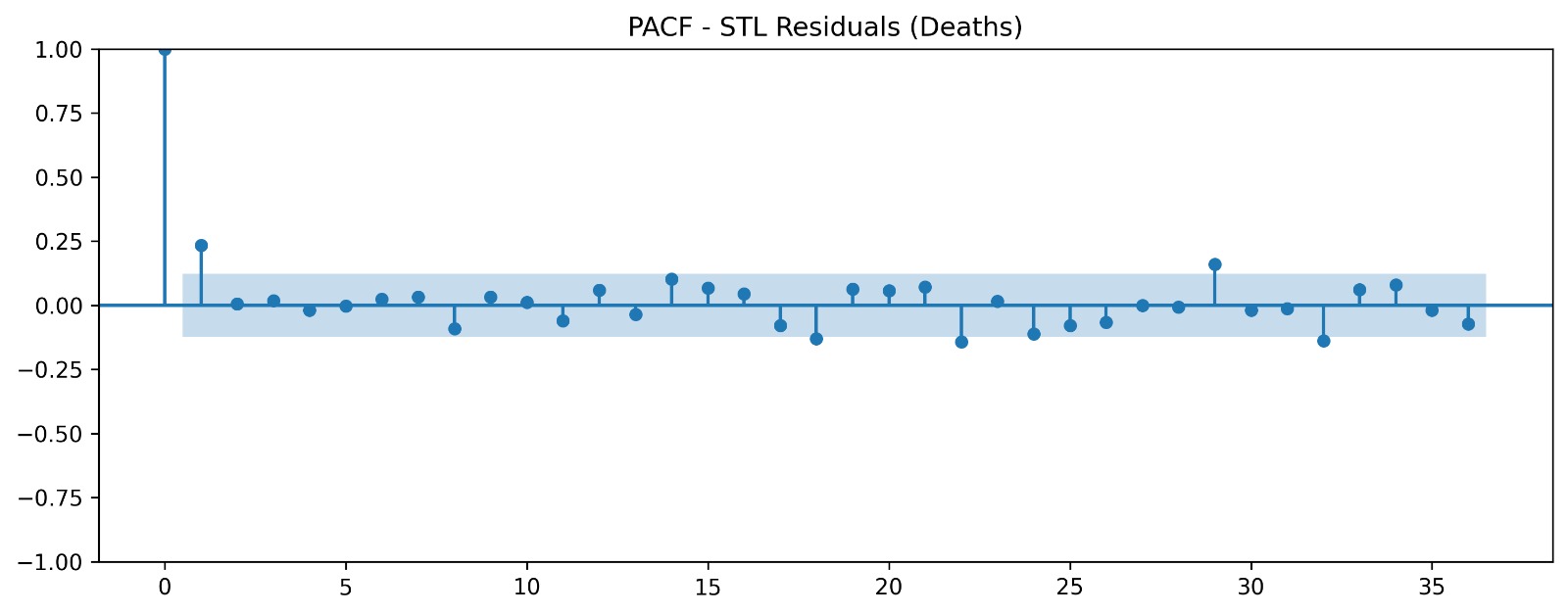


**Supplementary Figure 4.**PACF of STL residuals (VL deaths).
